# Targeting CTCF to Control Virus Gene Expression: A Common Theme amongst Diverse DNA Viruses

**DOI:** 10.3390/v7072791

**Published:** 2015-07-06

**Authors:** Ieisha Pentland, Joanna L. Parish

**Affiliations:** School of Cancer Sciences, University of Birmingham, Edgbaston, Birmingham, B15 2TT, UK; E-Mail: ixp370@bham.ac.uk

**Keywords:** CTCF, DNA virus, gene expression, cohesin, chromatin organization

## Abstract

All viruses target host cell factors for successful life cycle completion. Transcriptional control of DNA viruses by host cell factors is important in the temporal and spatial regulation of virus gene expression. Many of these factors are recruited to enhance virus gene expression and thereby increase virus production, but host cell factors can also restrict virus gene expression and productivity of infection. CCCTC binding factor (CTCF) is a host cell DNA binding protein important for the regulation of genomic chromatin boundaries, transcriptional control and enhancer element usage. CTCF also functions in RNA polymerase II regulation and in doing so can influence co-transcriptional splicing events. Several DNA viruses, including Kaposi’s sarcoma-associated herpesvirus (KSHV), Epstein-Barr virus (EBV) and human papillomavirus (HPV) utilize CTCF to control virus gene expression and many studies have highlighted a role for CTCF in the persistence of these diverse oncogenic viruses. CTCF can both enhance and repress virus gene expression and in some cases CTCF increases the complexity of alternatively spliced transcripts. This review article will discuss the function of CTCF in the life cycle of DNA viruses in the context of known host cell CTCF functions.

## 1. Introduction

CCCTC-binding factor (CTCF) is a ubiquitously expressed DNA binding protein that is highly conserved in bilaterian metazoans [1]. The protein contains 11 zinc fingers, 4 of which (zinc fingers 4–7) bind strongly to the core 12 base pair DNA sequence that is common in most CTCF binding sites [2,3]. It is thought that the remaining zinc fingers differentially bind to sequences up- and downstream of this central motif, allowing a large degree of flexibility and extension of the canonical binding site [4,5]. The exact number of CTCF binding sites within the human genome is not clear, but studies have shown up to 26,000 binding sites in human cells [3,6,7]. However, a more recent study has highlighted the potential for many more CTCF binding sites within the human genome (in the region of 300,000) whose occupancy depends on specific cell type and differentiation status [8]. CTCF binding appears to be enriched within intergenic spaces or in intronic regions of genes. Approximately 12% of CTCF binding sites are located within proximal promoters [8].

The association of CTCF with the human genome is important for genomic organisation and the control of gene expression. In particular, CTCF is essential for genomic imprinting, chromatin insulation, and transcriptional activation and repression. Furthermore, association of CTCF with specific regions of DNA has been linked to DNA looping, nucleosome organization, and the control of RNA Polymerase II (RNA Pol II) progression to co-ordinate co-transcriptional gene splicing events. Adding to the complexity of CTCF-mediated chromatin organization and the regulation of gene expression, DNA CpG methylation can influence CTCF DNA binding. Up to 41% of CTCF binding sites have been shown to be sensitive to CpG methylation [9]. Interestingly, inhibition of CTCF binding by DNA methylation has also been shown to alter gene-splicing events [10].

These important functions of CTCF in the control of host cell gene expression have been extensively reviewed elsewhere. We will therefore focus this article on emerging evidence that CTCF is utilised by diverse DNA viruses to control viral gene expression, genome organisation, virus replication and persistence.

## 2. CTCF-Mediated Virus Transcription Activation and Repression

The role of CTCF in transcriptional silencing and activation was first described in the 1990s [11,12,13]. These early studies demonstrated that CTCF binds to multiple sites within the *c-myc* gene promoter to repress transcription. A subsequent study showed that binding of CTCF to a core sequence located at the 5′ end of the chicken β-globin locus conferred strong enhancer blocking activity [14]. Furthermore, transcriptional control of the imprinted Igf2/H19 locus is mediated by CTCF binding to a differentially methylated region (DMR) within the imprinted control region (ICR). Methylation of the paternal ICR prevents CTCF binding, thus allowing downstream enhancers to act on the Igf2 promoter to facilitate Igf2 expression. Conversely, bound CTCF is present at the unmethylated maternal ICR, which blocks enhancers acting on the Igf2 promoter [15,16]. In this particular example, CTCF binding within the maternal allele blocks downstream enhancers from activating Igf2 expression by forming loops within the DNA that prevent interaction of the enhancer elements with the Igf2 promoter, thus promoting H19 expression from the maternal allele only [15,17,18].

The involvement of CTCF in the control of viral gene transcription has been demonstrated in several DNA viruses. In studies of Kaposi’s sarcoma-associated herpesvirus (KSHV), CTCF was shown to associate with several regions within the viral genome, the strongest of these binding regions was located at an intergenic site between the divergent ORF73 and K14 open reading frames (ORFs), which are active in the latent and lytic phases of the virus life cycle, respectively. The association of CTCF at this strong binding region occurs in a cell cycle dependent manner, specifically during mid-S phase to repress transcription of lytic genes [19]. Mutation of this CTCF binding cluster disrupted the recruitment of the cohesin complex (described in more detail below) and caused an increase in lytic gene expression due to derepression of the promoter which drives K14 expression [20]. This result was later confirmed by siRNA-mediated depletion of CTCF which showed a specific increase in the early lytic gene expression including K14 and ORF74, but a greater increase in ORF57 and ORF6 was noted [21]. This increase in lytic gene expression caused by depletion of CTCF resulted in a 20–25 fold increase in virion production, leading the authors to propose CTCF as a host cell restriction factor for KSHV lytic replication. Interestingly, it has also been shown that cohesin and CTCF binding at the promoter region of ORF50/RTA in KSHV represses ORF50 expression which is required for latent reactivation, providing further evidence that CTCF and cohesin behave as repressors of lytic transcription [22]. The idea that CTCF may function as a host cell restriction factor for viral infections may also be true for human papillomavirus (HPV) as mutation of a single conserved CTCF binding site in HPV type 18 results in an increase in viral oncoprotein E6 and E7 transcription, causing hyperproliferation of epithelial tissues [23]. In addition, CTCF has been proposed as a restriction factor for human cytomegalovirus (hCMV) infection as it plays a major role in limiting major immediate early (MIE) gene expression [24].

Interestingly, dynamic binding of CTCF at some sites within the KSHV genome has been demonstrated, and rather than a global eviction of CTCF upon lytic cycle activation, CTCF was gradually reduced at the majority of binding sites but maintained at others [21]. In this study, only a subset of lytic KSHV genes were transcriptionally enhanced upon CTCF knockdown, illustrating the use of site-specific CTCF binding. The authors conclude that this contributes to a mechanism whereby CTCF initially acts as a stimulator of lytic gene expression and then subsequently acts as an inhibitor of the lytic gene expression. Although the precise mechanism of this is unclear, it is interesting that CTCF binding is so intimately linked to the switch in latent to lytic gene expression in the KSHV life cycle.

In the Epstein-Barr virus (EBV) genome, CTCF binds to a site between the viral origin of replication (OriP) and the C promoter (Cp). Deletion of this CTCF binding site results in an increase in EBNA2 transcription levels, which is interesting considering EBV latency types are distinguished by their expression of EBNA2 levels. Furthermore, there was more total CTCF protein and mRNA detected in the type-I EBV cells compared to type-III. Overall, the presence of CTCF binding in EBV was shown to negatively affect transcription at Cp [25]. Additionally, when the functional CTCF binding site upstream of another EBV promoter termed Qp was abrogated there was a reduction in initiation of Qp transcription, and instead an alternative promoter (Fp) upstream was preferentially utilized causing overall disruption of latency transcript expression [26]. Mutation of a CTCF site positioned within the EBV LMP1 and LMP2A region also resulted in a decrease in LMP1 and LMP2A transcript expression [27].

There is also evidence to support a role for CTCF in regulating adenovirus replication and late gene expression as siRNA-mediated depletion of CTCF represses late gene expression but had no effect on early gene expression [28]. In addition, a recent study investigating the interaction of CTCF with human cytomegalovirus (hCMV) showed that CTCF binding in the first intron of the major intermediate early (MIE) gene caused repression of MIE gene expression, likely through a transcriptional mechanism believed to involve the manipulation of RNA pol II function [24].

## 3. Chromatin Barrier Formation

CTCF is the major transcriptional insulator in mammals and, by binding to specific sites within the cellular genome, creates a physical barrier between active and repressive chromatin, forming chromatin boundaries. Genome-wide chromatin immunoprecipitation (ChIP)-Seq analysis has demonstrated a significant enrichment of CTCF binding at regions located between active and repressive chromatin domains [7] and depletion of CTCF appears to cause heterochromatin spread [29,30]. The exact mechanism of how CTCF creates these boundaries remains elusive but it has been suggested that CTCF recruits a variety of binding partners and is differentially post-translationally modified to exert these effects (reviewed by [31]). During Herpes simplex virus type 1 (HSV-1) latent infection, the viral genome is organized in distinct transcriptionally repressed and active chromatin domains characterized by histone H3 hypo- and hyper-acetylation, respectively [32,33]. This separation of the active latency associated transcript (LAT) region and inactive ICP0 lytic promoter is controlled by a cluster of CTCF binding sites at the 3′ end of the LAT region [34,35]. During latent infection, CTCF binding the LAT region insulates the LAT enhancer from exerting effects on the adjacent ICP0 lytic promoter [34]. Binding of CTCF to the LAT region therefore creates a boundary which separates inactive chromatin in the ICP0 lytic gene region from an active LAT promoter region [35]. In the study by Tempera *et al.* [26] described above, mutation of the high affinity CTCF binding site immediately upstream of Qp in EBV resulted in reduced Qp activity but increased Cp and Fp activity. However, long-term culture of cells harboring this mutant bacmid resulted in no detectable Qp activity. This loss of Qp activity was associated with increased histone H3 lysine 9 trimethylation (H3K9me3) and CpG DNA methylation, indicating spread of inactive chromatin marks from the repressed region situated upstream of the mutated CTCF binding sites. Similarly, when the CTCF binding site in the EBV LMP1 and LMP2A region was abrogated there was a noticeable increase in CpG methylation at the corresponding promoter control sites [27]. Conversely, despite CTCF binding between the alternative replication initiation site (rep*) and Cp in the EBV genome of type III Burkitt's lymphoma (BL) cell lines, a high level of CpG methylation has been detected in this region, suggesting CTCF may not be able to prevent methylation across all target sequences [36].

These data elegantly highlight the ability of CTCF to function as a chromatin barrier within the context of a viral genome. Whether CTCF functions as a barrier to prevent the spread of inactive chromatin in small DNA viruses remains to be determined but it is interesting to note that loss of CpG methylation within the HPV16 genome has been demonstrated upon differentiation of host cells [37]. While the HPV life cycle does not have a lytic phase as such, capsid protein production is only induced in differentiated epithelial cells, suggesting that a molecular switch in early to late (capsid) gene expression controls completion of the virus life cycle. Whether this is linked to CTCF binding and epigenetic boundary formation that is altered upon host cell differentiation has yet to be determined.

## 4. Chromatin Loop Formation

One of the ways in which CTCF contributes to genome wide organization of chromatin is in the formation of loops that mediate long-range interactions between distant loci. Exactly how CTCF co-ordinates loop formation is not entirely understood. Evidence suggests that CTCF is able to form homodimers and complexes with proteins known to associate with insulator sites, such as the nucleolar protein nucleophosmin, providing evidence that loop formation is mediated by CTCF-associated protein complexes [38]. In addition, a number of studies have demonstrated co-localization of CTCF and the cohesion complex at thousands of genomic sites [39,40,41]. The cohesin complex is composed of four core subunits (Smc1, Smc3, Scc1/Rad21, Scc3), which form a ring-like structure that encircles DNA strands to facilitate sister chromatid cohesion during mitotic segregation (reviewed by [42]). Aside from its role in sister chromatid cohesion, there is evidence that co-localization of CTCF and cohesin at specific sites can facilitate the formation and stabilization of chromatin loops and thereby influence gene expression [43,44]. The role of CTCF in mediating long-range chromatin interactions at the Igf2/H19 locus (described above) and the β-globin locus has been well described. In the β-globin locus, multiple CTCF binding sites are known to interact [45] and a large (>50 kilobasepair) chromatin loop is formed between two CTCF binding sites [46], thus highlighting the possibility of CTCF mediated looping in the control of virus transcription.

In support of this hypothesis, CTCF has been shown to co-localize with cohesin in the KSHV episome on a region of the major latency control transcript during latent infection. Subsequent deletion of this CTCF binding site resulted in a loss of cohesin binding and a reduction in stable colony formation [20]. Co-incidentally, deletion of the CTCF binding site and loss of cohesin binding within the major latency control region of KSHV caused derepression of lytic gene expression, notably the K14/ORF74 transcript. This is similar to the role of Scc1 in transcriptional repression at the *c-myc* locus [20]. Interestingly, knockdown of Scc1 alone was shown to enhance KSHV virion production even more than CTCF knockdown alone, suggesting that cohesin has an even greater influence than CTCF on the control of lytic gene expression and that while these proteins co-locate in some areas of the viral genome, distinct localization of CTCF and cohesin may also occur [21]. Similarly, CTCF and cohesin are highly associated within the control region of the EBV latency membrane proteins LMP-1 and LMP-2A, with this co-occupancy strongly linked with DNA loop formation with the enhancer with the origin of replication (OriP) [27]. These studies highlight the role of CTCF and cohesin co-localization to regulate the complex process of gene expression and chromatin organization, through mediating DNA looping and structural changes of the chromatin.

To address the role of CTCF in mediating chromatin loop formation in EBV, chromatin conformation capture (3C) was used to reveal loop formation between CTCF binding sites at the type I latency promoter Cp and type III latency promoter Qp with the OriP enhancer. As predicted, loop formation was demonstrated between OriP and Qp during type I latency and between OriP and Cp during type III latency [47]. Subsequent deletion of the Qp CTCF binding site led to a loss in loop formation with OriP and a switch to Cp transcription instead. Furthermore, abrogation of CTCF binding at Cp led to a loss in both Qp-OriP and Cp-OriP loop associations, thus demonstrating a critical role for CTCF in this looping function and subsequent regulation of gene expression [47]. Similarly, CTCF-mediated loop formation between OriP and the LMP1/2 gene region of EBV has been described [48]. Abrogation of CTCF binding within OriP disrupts looping with LMP1 and LMP2A control regions leading to an increase in H3K9me3 and CpG methylation in the LMP1 and LMP2A promoter regions and upregulation of LMP2B transcription [27]. It is plausible that the ability of CTCF to confer loop formation between different sequences and regulatory elements can in part explain its varying effects on the control of gene transcription in the EBV life cycle. Furthermore, whilst a DNA looping mechanism may be advantageous for the genomic regulation of larger DNA viruses in order to assemble distal elements, the potential role of looping in much smaller DNA viruses such as adenovirus and HPV remains to be determined. It is likely however that DNA looping may not be required for smaller genome viruses and instead CTCF confers genomic regulation through alternative mechanisms. One potential counter-argument to this, however, has been provided by a study by Mehta and colleagues [49] in which the cohesion subunit Smc1 was shown to bind to the late gene region of the HPV31 genome in a CTCF-dependent manner. At least a subset of the Smc1 protein associated with HPV31 genomes is phosphorylated as part of the Ataxia-Telangiectasia Mutated (ATM)-dependent DNA damage response and is recruited to the HPV31 genome to support viral genome amplification. Whether recruitment of Smc1 to HPV genomes results in loop formation to regulate viral gene expression and/or genome amplification is an interesting question.

## 5. Nucleosome Positioning and RNA Polymerase II Progression

It has been demonstrated that CTCF can bind to the large subunit of RNA Pol II, which is needed for transcriptional initiation and elongation, and recruit RNA Pol II to target genes [50]. A recent study has also shown that, in co-operation with the general transcription factor TFII-I, CTCF influences the regulation of RNA pol II progression, downstream of recruitment to a pre-initiation complex. TFII-I and CTCF appear to facilitate the recruitment of the cyclin dependent kinase 8 (CDK8) complex, which phosphorylates RNA Pol II within its C-terminal domain at serine 5 to initiate mRNA synthesis [51]. In addition, binding of CTCF within exons has been shown to mediate RNA Pol II pausing and promote inclusion of weak upstream exons *via* co-transcriptional RNA splicing [10]. The potential of CTCF to regulate nucleosome positioning and RNA Pol II function as a mechanism for transcriptional control in DNA viruses has been investigated using a KSHV model system. Here, abrogation of CTCF binding in the first intron of the KSHV latency-associated multicistronic transcript resulted in an elevation or ORF73, ORF72, ORF71 and viral miRNA production but a concomitant decrease in K12 and ORF69 production, consistent with a previous study demonstrating loop formation between the CTCF binding site and the 3′ end of the K12 transcript [52,53]. Experiments also showed that CTCF was required for RNA Pol II programming, since loss of CTCF binding resulted in an increase in phosphorylation of RNA Pol II at serine 5 at the 5′ end of the latency transcript and a decrease in RNA Pol II S5 at the K12 ORF while causing the displacement of nucleosomes within the intron upstream of ORF73 [52]. It appears from these studies that rather than acting as a physical roadblock to RNA Pol II, CTCF may serve to regulate RNA Pol II within the intron upstream of ORF73 and mediate promoter selection by loop formation within the latency transcript unit.

Studies in KSHV have also shown that CTCF binding influences alternative splicing of the major latency transcripts [52], although an earlier study showed that CTCF recruitment to the KSHV genome was cell cycle dependent while alternative transcript splicing was not [19]. It has been proposed that CTCF binding to the EBV LMP region may facilitate RNA Pol II pausing and subsequent gene splicing events [27], although this is yet to be formally proven. Abrogation of CTCF binding by mutation of a binding site within the early gene region of the small DNA virus HPV type 18, in which transcripts are extensively alternatively spliced in order to generate multiple mRNAs and increase the repertoire of expressed proteins, causes a significant alteration of major splicing events important for the expression of early proteins [23]. However, the mechanistic underpinnings of CTCF function in the control of HPV transcript splicing remain to be determined.

## 6. Viral Genome Persistence

Cohesin and CTCF organize genomic DNA to create discrete nuclear regions to support efficient DNA replication [54]. Whether this is also true for viral DNA replication remains to be determined but the replication and stability of some DNA viruses is affected by CTCF recruitment. In some instances the mutation of specific CTCF binding sites has been shown to alter episomal maintenance. The CTCF binding site in Qp in the EBV genome and in the ORF73/LANA promoter region of the primate gamma-2 herpesvirus, herpesvirus saimiri (HVS) is important for maintaining viral episomes, as mutation to prevent CTCF binding led to a greater loss of episomes compared to the wild type genome for both of these viruses [26,55], although the effect of viral genome loss in both of these studies is more likely due to a reduction in the expression of viral transcripts required for latency. Similarly, disruption of CTCF binding sites in the KSHV latency control region also had a negative impact on stable episome maintenance during latency [20]. On the other hand lymphoblastoid cell lines (LCLs) harboring EBV bacmids with mutated CTCF binding sites at the LMP1 and LMP2 overlapping region revealed increased episome copy number compared to the cells containing wild type EBV bacmids [27]. While abrogation of CTCF binding within the early E2 ORF of HPV18 does not affect the maintenance of viral episomes (Pentland, Parish unpublished), mutating CTCF binding sites within the late L2 region of HPV31 caused a loss of viral episomes, indicating that CTCF may play a role in the replication or maintenance of HPV31 genomes [49]. These contrasting findings may demonstrate that episome maintenance is affected when specific CTCF binding sites are mutated in HPV and the location of these sites is likely to be important for determining the fate of viral maintenance. It has yet to be determined whether CTCF mediates integration of viruses into the host cell genome, or indeed whether CTCF participates in the reactivation of persistent, latent viral infections.

## 7. Conclusions

It is clear that CTCF plays an integral role in virus genome organization and gene regulation, in both large and small DNA viruses. Studies have demonstrated that CTCF organizes viral genomes by creating boundaries between active and inactive chromatin and can regulate viral gene expression by altering RNA pol II recruitment and progression, and nucleosome positioning. The co-localization of CTCF with cohesin in EBV and KSHV has highlighted the complex regulation of chromatin structure and the CTCF mediated DNA looping required to confer different genomic outcomes. The ability of CTCF to alter nucleosome positioning and RNA Pol II function adds further evidence to its role in altering gene splicing events and chromatin arrangement, in order to facilitate gene expression across the genome. It is unlikely that CTCF mediates separate functions alone but instead uses them in concert to co-ordinate complex genomic processes. It is intriguing that CTCF recruitment to some sites within viral genomes appears to be restrictive to virus replication, while recruitment to other sites results in activation of virus gene expression. The completion of a virus life cycle is without doubt a finely tuned balancing act and it is likely that DNA viruses have evolved to utilize CTCF to maintain this balance for efficient life cycle completion; CTCF thereby behaves as a restriction factor and an activating factor depending on virus life cycle stage and the specific context of CTCF binding. The continued study of viral interactions with CTCF will allow us to further understand the functions and implications of the vast array of CTCF-mediated events.
